# Effect of DPP-IV Inhibitors on Glycemic Variability in Patients with T2DM: A Systematic Review and Meta-Analysis

**DOI:** 10.1038/s41598-019-49803-9

**Published:** 2019-09-16

**Authors:** Subin Lee, Heeyoung Lee, Yoonhye Kim, EunYoung Kim

**Affiliations:** 1 Clinical Data Analysis and Evidence-based Research Lab. Department of Pharmaceutical Sciences, College of Pharmacy, Chung-Ang University Graduated School, Seoul, Republic of Korea; 2 Department of Clinical Pharmacy, College of Pharmacy, Gachon University, Incheon, South Korea; 3 Division of Licensing of Medicines and Regulatory Science, Graduate School Pharmaceutical Management, Chung-Ang University, Seoul, Republic of Korea

**Keywords:** Endocrinology, Outcomes research, Translational research

## Abstract

Glycemic variability (GV) has been an emerging target for preventing complications related to type 2 diabetes. For reducing GV, DPP-IV inhibitors have shown effectiveness compared to other oral anti-hyperglycemic drugs (OADs), but systematic evaluation has yet to be existed. A systematic review and meta-analysis of randomized controlled trials (RCTs) were performed to evaluate the effect of DPP-IV inhibitors compared with other OADs, on GV as measured by mean amplitude of glycemic excursions (MAGE). Searches were conducted using Pubmed, EMBASE, and the Cochrane Library, from which eligible studies were retrieved; seven RCTs were included in the analysis. DPP-IV inhibitors were found to significantly reduce MAGE compared to other OADs (mean difference = −14.61; 95% CI = −19.00 to −10.21; *p* < 0.0001) without significant heterogeneity among sulfonylureas (mean difference = −14.93; 95% CI = −21.60 to −8.26; *p* < 0.0001). Initial combination therapy with DPP-IV inhibitors more effectively reduced MAGE than stepwise add-on therapies (*p* = 0.006), although no differences in MAGE were found based on HbA1c values. These findings indicate that DPP-IV inhibitors are promising alternatives for reducing GV in type 2 diabetes patients. However, further studies utilizing larger numbers of patients and longer-term follow-ups are needed.

## Introduction

Unregulated hyperglycemia in diabetic patients is associated with increased diabetic complications such as cardiovascular disease^1^ that has resulted in an additional 2.2 million deaths^2^. To reduce such risks, glucose variability (GV), the term for glycemic fluctuation, has emerged as an important clinical predictor and an essential target for dysglycemia treatment in diabetic patients^3^. Although glycated hemoglobin (HbA1c) is still a standard clinical marker for long-term glycemia, a reduction in blood glucose fluctuations has been significantly correlated with reduced morbidity and mortality in diabetic patients.

Dipeptidyl-peptidase IV (DPP-IV) inhibitors have been found to reduce blood glucose fluctuations and improve glycemic control in type 2 diabetes mellitus (T2DM) patients. Pharmacologically, DPP-IV inhibitors enhance glucagon-like peptide-1 (GLP-1) preservation and expansion of β-cell mass through the inhibition of apoptotic pathways, improving blood glucose control without inducing hypoglycemia^4,5^. Since GV is highly correlated with pancreatic β-cell dysfunction even in patients whose T2DM is well-controlled^6^, DPP-IV inhibitors are considered effective for reducing GV. In previous studies, however, different pharmacokinetic profiles of DPP-IV inhibitors^7^ and other types of oral antihyperglycemic drugs (OADs)^8^ revealed significant efficacy discrepancies among them in glycemic control. Furthermore, for reducing GV, clinical trials conducted with DPP-IV inhibitors provided discriminated outcomes of efficacy compared with other OADs^9,10^. Although some systematic reviews and meta-analyses studies^11,12^ have found an advantage for DPP-IV inhibitors over existing therapies with oral antidiabetic compounds, they did not show a specific beneficial impact of DPP-IV inhibitors on reducing GV in T2DM patients. The current study, a systematic review and meta-analysis of pooled outcome data from available randomized controlled trials (RCTs), was performed to provide more evidence with higher statistical power regarding the impact of DPP-IV inhibitors on GV in patients with T2DM.

## Results

### Study selection

**Figure 1:**
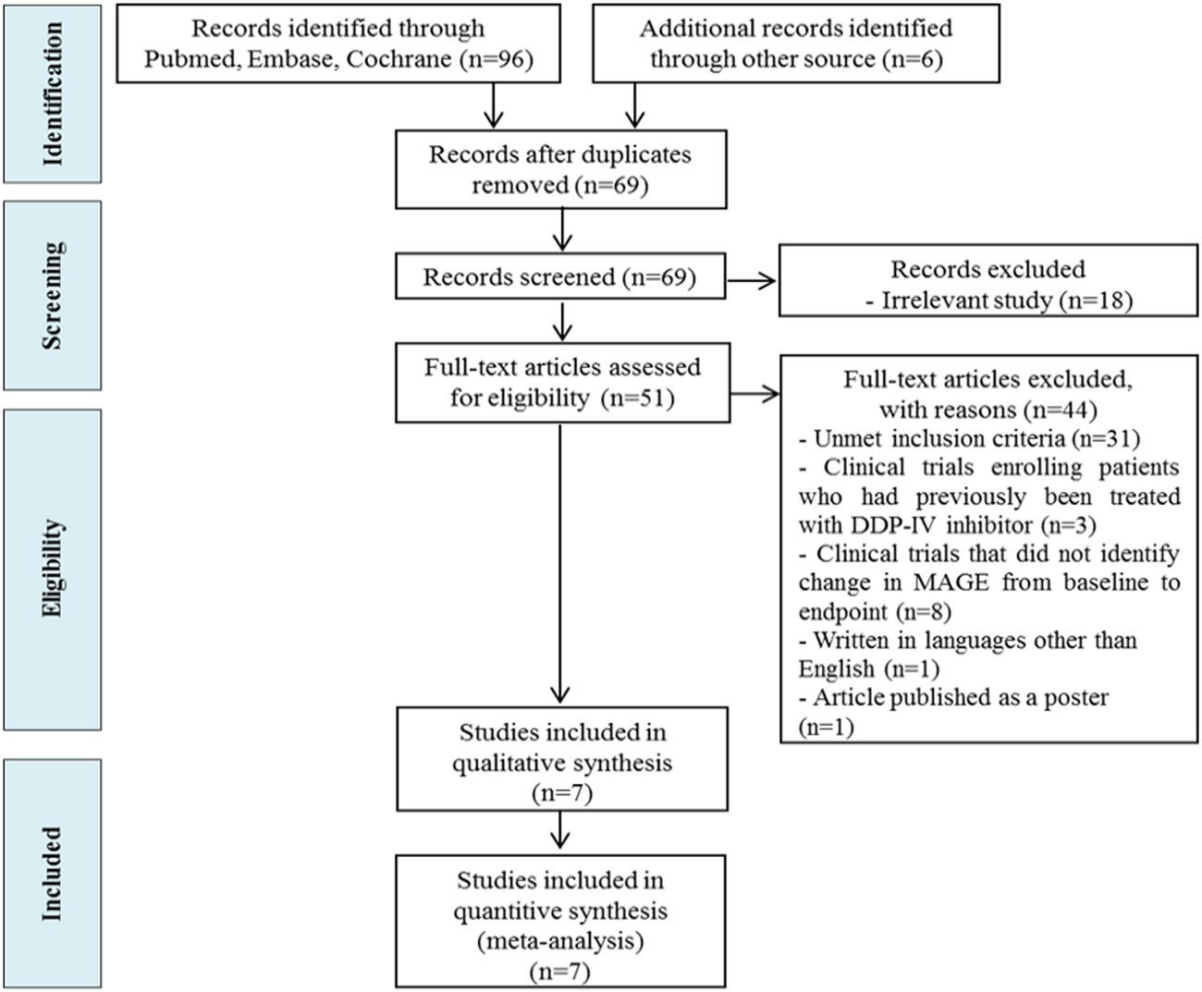
Flowchart of study identification and selection.

A comprehensive search identified 102 potentially relevant articles from PubMed, EMBASE, and the Cochrane Library. After full-text reviews of these articles, this was narrowed to 51 articles, from which seven articles were included in the analysis (Fig. 1).

### Study description

Characteristics of the seven studies^9,10,13–17^ included in the analyses are shown in Table 1. The total number of included patients was 304, and all were Asian T2DM patients. Five of the seven studies^9,10,13,15,16^ were conducted in the Republic of Korea, one^14^ in China, and one^17^ in Japan. Sample sizes ranged from 25 to 62 individuals. Two studies^10,14^ were performed in patients who underwent initial combination therapy combining DPP-IV inhibitors or other OADs with metformin. Four studies^9,13,15,16^ added DPP-IV inhibitors or other OADs to metformin as stepwise add-on therapy for treating T2DM patients. However, Suzuki *et al*.^17^ treated drug-naïve T2DM patients with sitagliptin or glibenclamide alone. The timing of mean amplitude of glycemic excursion (MAGE) measurements by continuous glucose monitoring system (CGMS) was commonly at 2–3 days at the baseline and at the end of treatment in the included studies. Six of the included studies^9,10,13,14,16,17^ were parallel designs, and one^15^ was a crossover study.

**Table 1:** Characteristics of the included studies.

Study name	Publication year	Country	No. of patients (Intervention /Comparator)	DDP-IV inhibitors	Other OADs	Therapy type	Study design	Follow-up period (weeks)	Timing of MAGE measurements
Kim HS *et al*.^13^	2013	Republic of Korea	16/17	Sitagliptin	Glimepiride	Add-on	Parallel	4	For 3 successive days at baseline and at the end of follow-up
Xiao *et al*.^14^	2016	China	23/18	Sitagliptin	Glimepiride	Combination	Parallel	24	For 72 hours, at baseline, 4, 8, 12, and 24- weeks
Kim NH *et al*.^9^	2017	Republic of Korea	14/11	Vildagliptin	Pioglitazone	Add-on	Parallel	16	For 3 consecutive days at baseline and at the end of follow-up
Park KS *et al*.^15^	2017	Republic of Korea	16/16	Vildagliptin	Glimepiride	Add-on	Crossover	12	For 3 consecutive day measurements, at baseline and at the end of follow-up
Kim G *et al*.^16^	2017	Republic of Korea	17/17	Vildagliptin	Glimepiride	Add-on	Parallel	12	For 3 consecutive day measurements, at baseline and at the end of follow-up
Park SE *et al*.^10^	2017	Republic of Korea	24^a^, 21^b^/17	Gemigliptin Sitagliptin	Glimepiride	Combination	Parallel	12	For 3 consecutive day measurements at baseline and at the end of follow-up
Suzuki *et al*.^17^	2018	Japan	26/26	Sitagliptin	Glibenclamide	Mono	Parallel	2	For 3 consecutive days at baseline and at the end of follow-up

Values are presented as mean ± SD, ^a^Gemigliptin + Metformin group, ^b^Sitagliptin + Metformin group.

Baseline characteristics of body mass index (BMI), HbA1c, age, and MAGE are presented in Table 2. All included studies reported no significant differences in baseline characteristics of BMI and HbA1c between intervention and comparison groups.

**Table 2:** Baseline characteristics of BMI, HbA1c, age, and MAGE.

Study name	BMI		HbAlc		Mean Age, y		MAGE	
	Intervention group	Comparator group	Intervention group	Comparator group	Intervention group	Comparator group	Intervention group	Comparator group
Kim HS *et al*.^13^	25.2 ± 2.2	25.9 ± 3.4	7 ± 0.5	7.3 ± 0.4	59.6 ± 6.7	55.8 ± 6.6	88.2 ± 18	102.6 ± 27
Xiao *et al*.^14^	28.34 ± 3.81	27.92 ± 3.87	7.32 ± 1.01	7.27 ± 1.15	68.7 ± 6.3	69.1 ± 6.5	145.08 ± 21.24	148.86 ± 21.78
Kim NH *et al*.^9^	25.8 ± 2.7	27.4 ± 4.3	7.2 ± 0.2	7.4 ± 0.4	59.9 ± 10.2	52.1 ± 11.1	93.8 ± 38	98.7 ± 31.8
Park KS *et al*.^15^	25.5 ± 4.1	25.5 ± 4.1	8.4 ± 0.9	8.4 ± 0.9	60.0 ± 9.6	60.0 ± 9.6	101.22 ± 26.62	101.22 ± 26.62
Kim G *et al*.^16^	26.6 ± 2.6	25.2 ± 3.9	7.6 ± 0.7	7.5 ± 0.5	55.6 ± 8.4	56.3 ± 5.7	97.4 ± 35.8	85.4 ± 31.6
Park SE *et al*.^10^	26.6 ± 3.9^a^ 25.9 ± 3.5^b^	26.0 ± 3.3	9.5 ± 1.8^a^ 9.1 ± 1.2 ^b^	9.7 ± 1.9	48.9 ± 10.7^a^ 49.6 ± 10.0^b^	51.5 ± 13.0	103 ± 27 ^a^ 96 ± 30 ^b^	95 ± 40
Suzuki *et al*.^17^	24.4 ± 2.1	24.6 ± 3	7.7 ± 0.5	7.9 ± 0.6	60.2 ± 8.4	59.5 ± 10.7	111.78 ± 38.16	110.70 ± 18.54

Values are presented as mean ± SD, ^a^Gemigliptin + Metformin group, ^b^Sitagliptin + Metformin group.

### Overall comparison of DPP-IV inhibitors to other OADs

**Figure 2:**
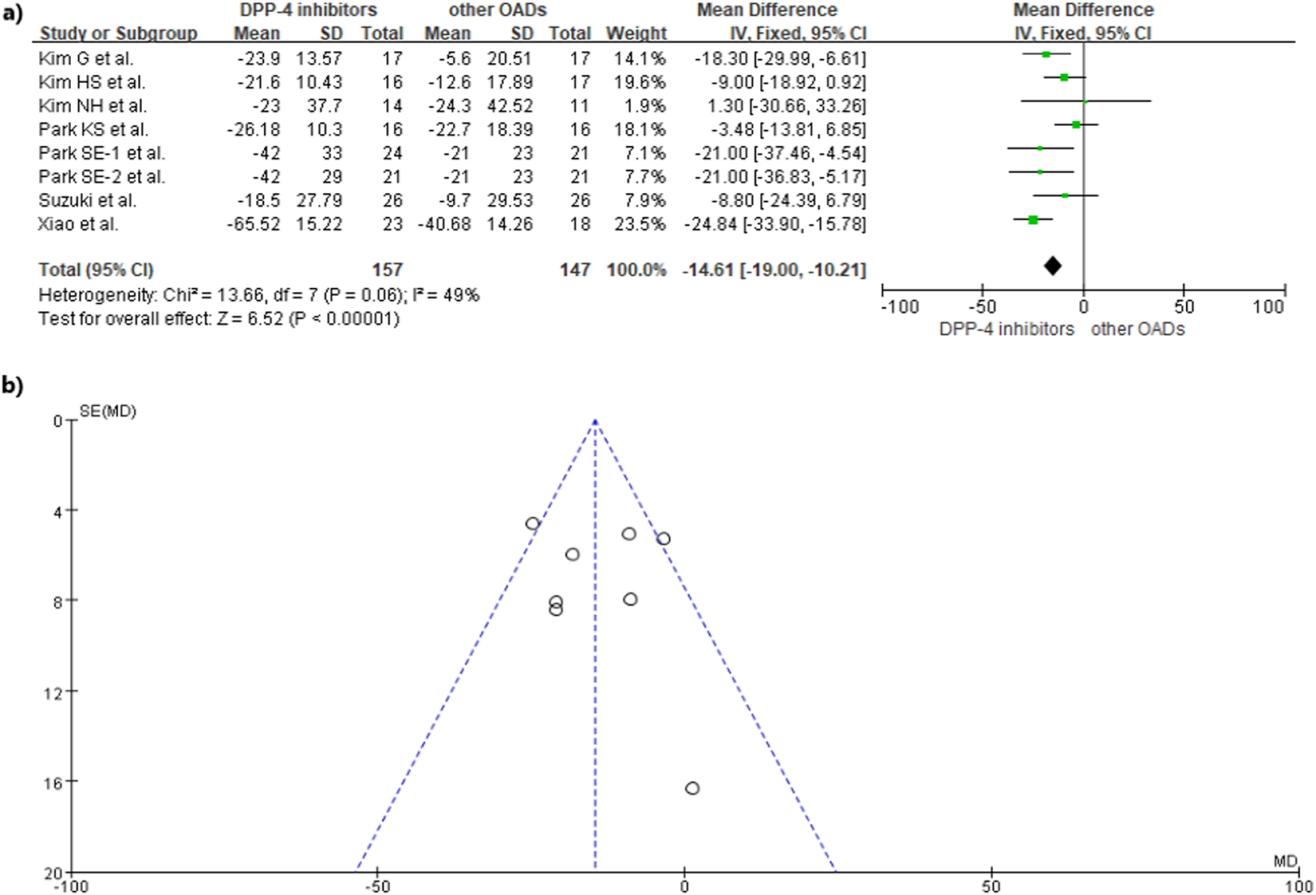
(**a)** Forest plot of overall differences in MAGE between DPP-IV inhibitors and other OADs (**b**) Funnel plot for reporting publication bias.

**Figure 3:**
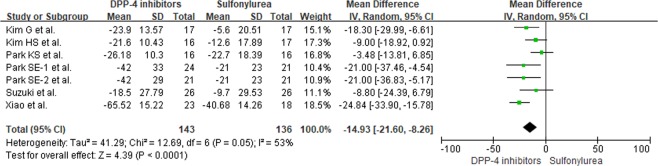
Differences in MAGE between DPP-IV inhibitors and sulfonylureas.

All included studies^9,10,13–17^ were analyzed to compare DPP-IV inhibitors to other OADs. Park SE *et al*.^10^ reported on two types of DPP-IV inhibitors, gemigliptin and sitagliptin, so data was separately extracted from each component of the study. An overall comparison between DPP-IV inhibitors and other OADs in reducing GV showed that DPP-IV inhibitors significantly reduced MAGE (mean difference (MD) = −14.61; 95% CI = −19.00 to −10.21; *p* < 0.0001, Fig. 2(a)). In addition, a comparison between DPP-IV inhibitors and sulfonylureas also showed a more significant reduction in MAGE for DPP-IV inhibitors in T2DM patients (MD = −14.93; 95% CI = −21.60 to −8.26; *p* < 0.0001, Fig. 3). No significant heterogeneity was observed.

### Comparing DPP-IV inhibitors to other OADs according to type of therapy

**Figure 4:**
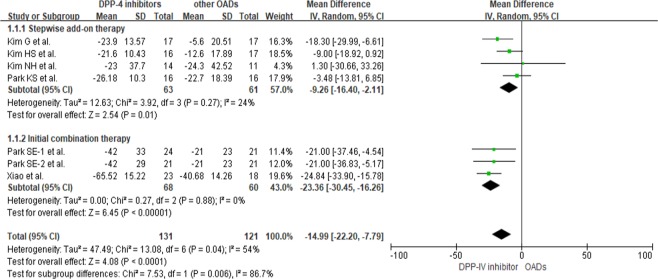
Differences in MAGE between DPP-IV inhibitors and other OADs according to types of therapy.

Six studies^9,10,13–16^ were analyzed to compare the efficacies of DPP-IV inhibitors and other OADs based on the type of therapy. Four studies^9,13,15,16^ were conducted on stepwise add-on therapy, and two^10,14^ on initial combination therapy (Fig. 4). Both add-on and combination therapies showed a significant reduction in MAGE (*p* < 0.05). However, initial combination therapy reduced MAGE to a greater degree (MD = −23.36; 95% CI = −30.45 to −16.26; *p* < 0.00001) than stepwise add-on therapy (MD = −9.26; 95% CI = −16.40 to −2.11; *p* = 0.01).

### Comparing DPP-IV inhibitors to other OADs according to HbA1c

**Figure 5:**
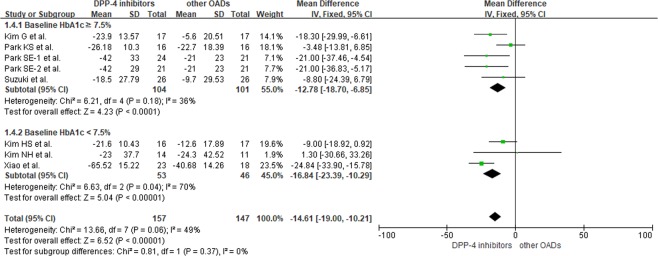
Differences in MAGE between DPP-IV inhibitors and other OADs according to HbA1c level.

DPP-IV inhibitors significantly reduced MAGE in comparison to other OADs in patients with a baseline HbA1c greater than 7.5% (MD = −12.78; 95% CI = −18.70 to −6.85; *p* < 0.0001) and in patients with a baseline HbA1c of less than 7.5% (MD = − 16.84; 95% CI = − 23.39 to −10.29; *p* < 0.00001). For overall reduction of MAGE, there was no significant difference between the two subgroups (Fig. 5).

### Risk of bias and quality of evidence

Assessments of risk of bias among included studies are shown in Supplementary Figure S1. All included studies showed a low risk of bias in attrition and reporting. Other than the study by Park SE *et al*.^10^ where selection and detection bias risk was identified as low, risks for selection and detection biases were unclear for the remaining studies. In this review, no publication bias was uncovered (*p* = 0.698, Fig. 2(b)). Table 3 illustrates evidence quality using the Grading of Recommendations, Assessment, Development, and Evaluation (GRADE) approach, with respect to the effects of DPP-IV inhibitors compared to other OADs in reducing MAGE.

**Table 3:** Summary of findings for MAGE reduction compared DPP-IV inhibitors to other OADs based on the GRADE approach.

Outcome	No. of Participants (studies)	Limitation	Inconsistency	Indirectness	Imprecision	Publication bias	Mean difference* (95% CI)	Quality of Evidence
Overall	304 (8)	No serious	No serious	No serious	No serious	Undetected	−14.61 (−19.00, −10.21)	⊕⊕⊕⊕ High
Compared to sulfonylurea	279 (7)	No serious	No serious	No serious	No serious	Undetected	−14.93 (−21.60,−8.26)	⊕⊕⊕⊕ High
***According to types of therapy***								
Stepwise addition therapy	124 (4)	No serious	No serious	No serious	No serious	Undetected	−9.26 (−16.40, −2.11)	⊕⊕⊕⊕ High
Initial combination therapy	128 (3)	No serious	Serious	No serious	No serious	Undetected	−23.36 (−30.45, −16.26)	⊕⊕⊕◯ Moderate
***According to HbA1c***								
HbA1c ≥7.5%	205 (5)	No serious	No serious	No serious	No serious	Undetected	−12.78 (−18.70, −6.85)	⊕⊕⊕⊕ High
HbA1c ≤7.5%	99 (3)	No serious	No serious	No serious	No serious	Undetected	−16.84 (−23.39, −10.29)	⊕⊕⊕⊕ High

CI, Confidence Interval; ICU, intensive care unit; MICU, medical intensive care unit; PICU, pediatric intensive care unit; observational, observational study; RCT, randomized controlled trial. ⊕ = attainment of Grading of Recommendations, Assessment, Development, and Evaluation criteria. *p < 0.05.

### Meta-regression analysis

Baseline HbA1c (slope = −4.03; 95% CI = −20.52 to 12.46; *p* = 0.63) and age (slope = −0.58; 95% CI = −2.44 to 1.28; *p* = 0.54) did not significantly influence the effect of DPP-IV inhibitors on MAGE reduction (Supplementary Fig. S2). However, BMI significantly influenced on MAGE reduction by DPP-IV inhibitors (slope = −11.42; 95% CI = 2.74 to −16.79; *p* < 0.0001).

## Discussion

The present study conducted a systematic review and meta-analysis to evaluate the efficacy of DPP-IV inhibitors in comparison to other OADs in reducing GV in patients with type 2 diabetes. The present study showed DPP-IV inhibitors significantly reduced MAGE, a marker of daily blood GV, in comparison with other OADs. In patients with T2DM, blood glucose control is essential preventing diabetic complications, and reducing GV has been associated with a decrease in the endothelial cell damage that leads to atherosclerotic cardiovascular disease (ASCVD)^18,19^, the leading cause of death in people with T2DM^20^. According to Dore *et al*.^21^, combined therapy of metformin with DPP-IV inhibitors improved endothelial dysfunction and systolic blood pressure (SBP) in T2DM patients (*p* < 0.05). Thus, findings of the current study intensify previous evidence for recommending DPP-IV inhibitors for T2DM patients at high risk for developing cardiovascular disease^22^.

In addition, the present study showed DPP-IV inhibitors more significantly reduced MAGE as compared to sulfonylureas. Sulfonylureas are the most common OAD due to their low-cost and high glucose-lowering capacity^23^; these compounds stimulate pancreatic β-cells to release insulin in a glucose-independent manner^24^. Prior studies have also demonstrated that including sulfonylureas in glucose-lowering regimens contributed to a decrease in microvascular complications^25,26^. However, concerns about the weight gain and increased risk for hypoglycemia associated with sulfonylureas use in T2DM patients led to an increased administration of DPP-IV inhibitors^27^, which also enhance insulin secretion. GV refers to the changes in blood glucose concentration from peaks to minimum values. Factors that might contribute to GV deterioration include decreased endogenous insulin secretion, deficiency in the relevant suppression of glucagon, and the use of hypoglycemic agents^27^. Different from insulin or sulfonylureas stimulating insulin secretion from pancreatic β cells, DPP-IV inhibitors increase GLP-1 levels, promoting insulin secretion and suppressing glucagon secretion in blood-glucose dependent manner. Because of the mechanism of action of DPP-IV inhibitors, it is presumed that DPP-IV inhibitors contribute to GV reduction^27^. With similar insulin secretion induction capacity, intrinsic pharmacological differences of DPP-IV inhibitors might contribute to a greater reduction in MAGE than sulfonylureas; this was suggested by findings from the present study. For T2DM characterized by lower insulin secretion, less adiposity, and less insulin, DPP-IV inhibitors may be a preferable treatment option^4,28^ to sulfonylureas.

The present study demonstrated that initial combination therapy with DPP-IV inhibitors was more effective in reducing MAGE than stepwise add-on therapy. Limited glycemic target achievement is recommended for treating T2DM patients, whether it be with monotherapy, stepwise add-on therapy, or initial combination therapy^22^. Currently, there is insufficient evidence for the American Diabetes Association (ADA) and the European Association for the Study of Diabetes (EASD) to recommend stepwise add-on therapy over initial combination therapy for patients with T2DM^22^. However, when monotherapy is unable to fully achieve glycemia target in newly diagnosed T2DM patients, initial combination therapy has been suggested as an alternative approach^29^. Furthermore, treatment intensification occurs more rapidly in higher risk patients relative to those with lower HbA1c; as such, many patients do not receive additional treatment to enhance glycemic control in a timely manner^30^. According to Brown and Nichols *et al*.^31^, an HbA1c greater than or equal to 9% usually induced the addition of glycemic lowering therapy to T2DM patients. Also, Cheung *et al*.^32^ reported 66% of patients with poorly controlled T2DM (HbA1c ≥ 8%) received a therapy amendment within six months. Prior meta-analyses^29,33^ have consistently advocated initial combination therapy, rather than stepwise add-on therapy, with DPP-IV inhibitors to reduce HbA1c in T2DM patients. For reducing GV, the current analysis showed that initial combination therapy with DPP-IV inhibitors is more effective than add-on therapy. Thus, for enhancing glycemic lowering therapy, initial combination therapy with DPP-IV inhibitors should be the first consideration for reducing GV in people with T2DM.

The present study also showed DPP-IV inhibitors significantly reduced MAGE in comparison with other OADs in T2DM patients regardless of baseline HbAlc level. HbA1c is currently the gold standard index for glycemic control; however, the effects of daily blood glucose fluctuations are not necessarily reflected in HbA1c levels because their probable relationship to β cell dysfunction^6^. Previous studies^34,35^ have asserted that HbA1c was not significantly related to glycemic control status measured by GV, and the current study also showed the efficacy of DPP-IV inhibitors did not vary according to HbA1c level. Since HbA1c is of limited value in assessing GV^36^, treatment by DPP-IV inhibitors should be administered to reduce glucose fluctuation in all T2DM patients regardless of HbA1c value.

This study had its limitations. First, studies included in the analysis evaluated the effect of DPP-IV inhibitors on GV over a limited duration. The long-term effects of DPP-IV inhibitors on GV need to be further investigated in clinical studies with more patients and longer treatment durations. Second, because the patients included in this study were all Asian, these results may not extend to other ethnic groups. Third, the comparison groups were mostly prescribed sulfonylureas rather than other anti-hyperglycemic drugs such as α-glucosidase inhibitors, or SGLT-2 inhibitors, among others, also limiting the generalization of these results. In addition, our study demonstrated BMI significantly influenced the MAGE reduction by meta-regression analysis without impacts of HbA1c and age. Although ADA/EASD supported to use DPP-IV inhibitors as adjunctive treatment for limiting weight gain^22^, our study included limited number of studies to confirm the effects of BMI to MAGE reduction. Thus, more future studies are warranted to evaluate the correlation between BMI and MAGE. Finally, a cost-benefit analysis comparing DPP-IV inhibitors to other OADs was outside the scope of this study; however, the ADA and EASD have indicated that DPP-IV inhibitors are relatively inexpensive^22^. DPP-IV inhibitors could be a good option to reduce socioeconomic barriers to accessing glucose lowering medications.

To our knowledge, this is the first systematic review and meta-analysis that evaluates the efficacy of DPP-IV inhibitors compared to other OADs in reducing MAGE. Reducing glucose fluctuation measured by MAGE is essential for lowering the risk of cardiovascular disease in T2DM patients. As best treatment options and glycemic control status are individual considerations when treating patients in practice, DPP-IV inhibitors are good options for many T2DM patients.

## Conclusion

The current study showed DPP-IV inhibitors significantly reduced GV compared to other OADs in T2DM patients. Reduction of GV was more significant in those treated with initial combination therapy, but no differences were observed among patients with different HbA1c baselines. DPP-IV inhibitors could be good treatment alternatives for reducing complications of T2DM caused by GV. More clinical trials should be performed in the future in other settings involving more T2DM patients to support the evidence presented here for the efficacy of DPP-IV inhibitors.

## Methods

This study was performed according to PRISMA statement recommendations^37^.

### Data sources and search strategy

PubMed, the Cochrane Library, and EMBASE were used to search the articles. Each database search was extended through June 2018, using the search keywords “glycemic variability,” “glycemic fluctuations,” “mean amplitude of glycemic excursions” in combination with “Diabetes Mellitus, type 2” and “dipeptidyl-peptidase IV inhibitor” combined with relevant MeSH terms and the substance names of marketed DPP-IV inhibitors. The references of the selected articles and the *Journal of Diabetes and its Complications* (2010) were also manually searched to retrieve additional studies. Two investigators independently evaluated the identified articles. Disagreements between investigators were resolved by discussion.

### Study selection

Two independent investigators first evaluated titles and abstracts of collected literature to find potentially related articles. All RCTs enrolling T2DM patients were selected. For inclusion, study treatment periods needed to be one week or longer, and compare DPP-IV inhibitors to other OADs. Studies calculating MAGE proposed by Service *et al*.’s^38^ and providing MAGE outcomes assessed by CGMS were included in the current analysis. Studies enrolling nondiabetics, type 1 diabetics, or patients previously treated with DPP-IV inhibitors were excluded. Animal studies, studies with a sample size of fewer than five patients, and articles written in languages other than English were excluded. Articles published as abstracts only were also excluded.

### Data extraction and quality assessment

Data extracted from the retrieved articles included publication year, study design, types of therapies, type of DPP-IV inhibitors, and type of comparison, except for DPP-IV inhibitors, sample size, age of study population, HbA1c, BMI, and MAGE.

Net change in MAGE was calculated as the difference between pre- and post-treatment values. Two investigators extracted the data and assessed the internal validity and quality of the retrieved articles. The study quality of RCTs was assessed using the Risk of Bias assessment tool^39^ developed by the Cochrane Collaboration. Confidence levels were evaluated for effect estimates for each outcome, and evidence quality was valued as high, moderate, low, or very low by the GRADE approach that examines study limitations, inconsistency, indirectness, imprecision, and publication bias^40^. Disagreements among investigators were resolved by discussion.

### Data synthesis and analysis

The present study evaluated overall differences in MAGE between DPP-IV inhibitors and other OADs in T2DM patients. For subgroup analyses, evaluation of MAGE changes between DPP-IV inhibitors and other OADs according to therapy type and HbA1c value (≥7.5% vs. <7.5%) was performed. HbA1c was divided by baseline level at 7.5% according to the amplitude of correlation between HbA1c and MAGE^41^. Also, MAGE changes were evaluated by comparing DPP-IV inhibitors and sulfonylureas after in T2DM patients. The overall effect size was presented as MD, and the 95% CI for the studies was derived with meta-analysis software; a *p-*value of less than 0.05 was considered statistically significant.

I^2^ statistics were used to analyze the significance of the heterogeneity among studies. Values of 25%, 50%, and 75% suggested low, medium, and high heterogeneity, respectively^42^. In addition, funnel plots were created, and Egger’s linear regression tests were conducted to test for publication bias^43^, with a *p-*value of less than 0.05 indicating the presence of bias. Also, a meta-regression on the DPP-IV inhibitors group was performed to examine contributions of patients’ clinical characteristics such as BMI, HbA1c, and age at baseline, on the effect of these inhibitors on MAGE. Meta-regression was used to examine the quantitative influence of study characteristics on the effect size^44^. The overall effect size was analyzed as net change in MAGE calculated as the difference between pre- and post-treatment, with BMI, HbA1c, and mean age at baseline included as covariates. Lastly, a meta-analysis for changes in HbA1c between pre- and post-treatment was performed, excluding the articles with a treatment period of less than 12 weeks, as a short-term period is insufficient to assess HbA1c. Data were analyzed using RevMan version 5.3. (Copenhagen: The Nordic Cochrane Centre, The Cochrane Collaboration, 2014) and Comprehensive Meta-Analysis (Biostat, Englewood, USA).

## Supplementary information

Risk of bias

Meta-regression

## Data Availability

The datasets analyzed during the current study are available from the corresponding author on reasonable request.
