# Supplementary material for: Effect of DPP-IV Inhibitors on Glycemic Variability in Patients with T2DM: A Systematic Review and Meta-Analysis
Source: Sci Rep. 2019 Sep 16;9:13296. doi: 10.1038/s41598-019-49803-9 (PMC6746852; doi:10.1038/s41598-019-49803-9)
Supplement: Supplementary file 1 — Risk of bias [file 41598_2019_49803_MOESM1_ESM.pdf]

Effect of DPP-IV Inhibitors on Glycemic Variability in Patients with T2DM: A Systematic Review and Meta-Analysis

Subin Lee, Heeyoung Lee, Yoon Hye Kim, Eunyoung Kim

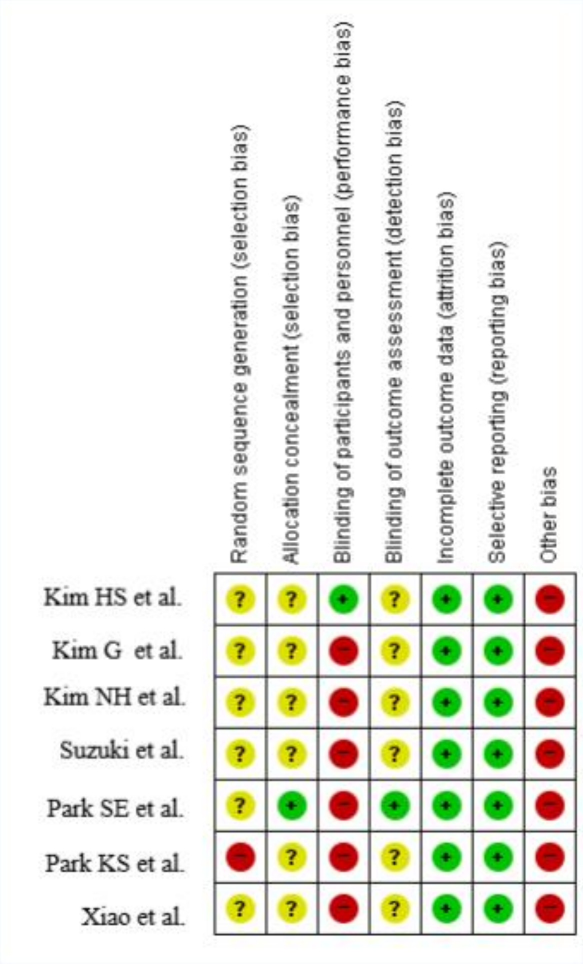
Random sequence generation (selection bias)
Allocation concealment (selection bias)
Blinding of participants and personnel (performance bias)
Blinding of outcome assessment (detection bias)
Incomplete outcome data (attrition bias)
Selective reporting (reporting bias)
Other bias
Kim HS et al.
Kim G et al.
Kim NH et al.
Suzuki et al.
Park SE et al.
Park KS et al.
Xiao et al.
