# Supplementary material for: Effect of DPP-IV Inhibitors on Glycemic Variability in Patients with T2DM: A Systematic Review and Meta-Analysis
Source: Sci Rep. 2019 Sep 16;9:13296. doi: 10.1038/s41598-019-49803-9 (PMC6746852; doi:10.1038/s41598-019-49803-9)
Supplement: Supplementary file 2 — Meta-regression [file 41598_2019_49803_MOESM2_ESM.pdf]

Effect of DPP-IV Inhibitors on Glycemic Variability in Patients with T2DM: A Systematic Review and Meta-Analysis

Subin Lee, Heeyoung Lee, Yoon Hye Kim, Eunyoung Kim

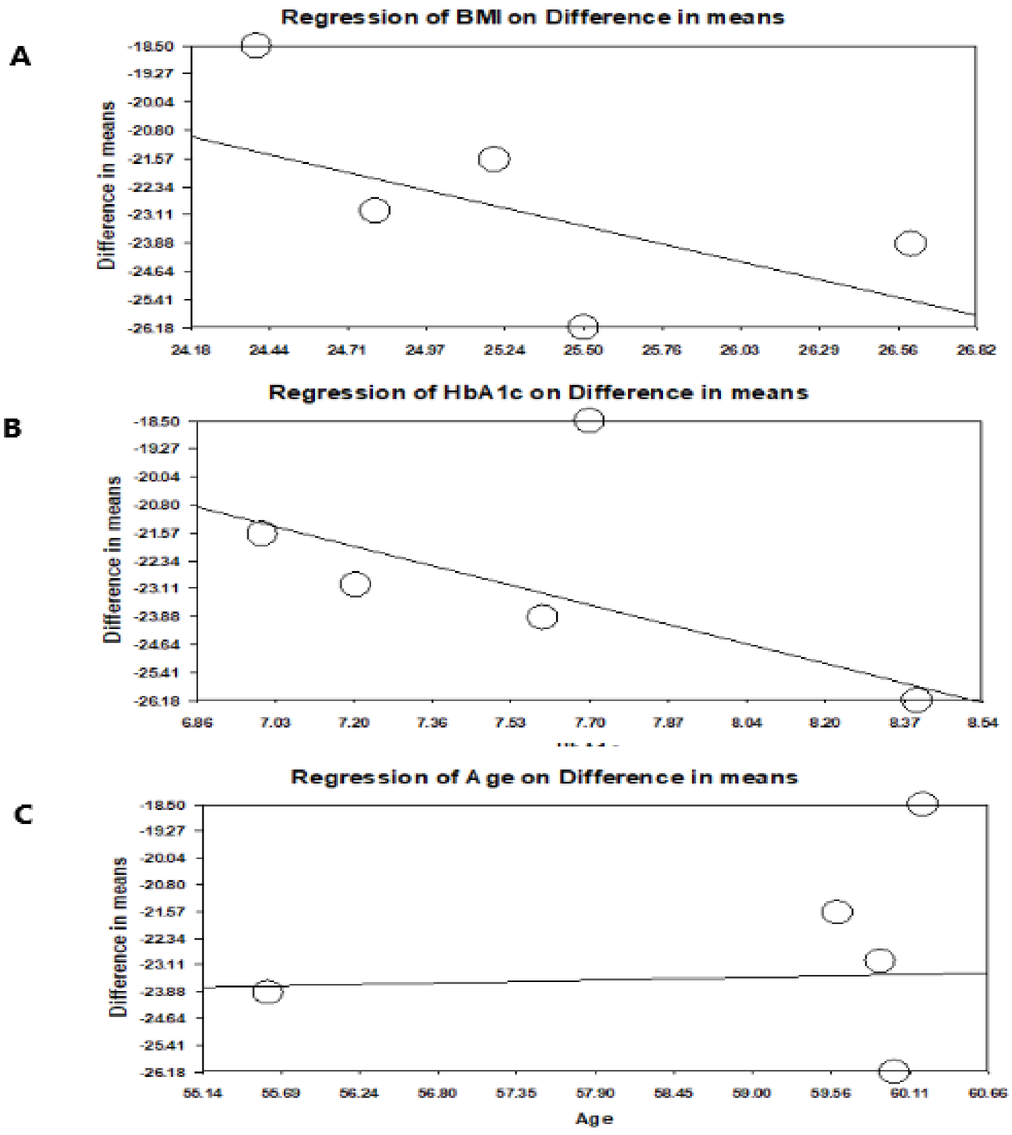
A
Regression of BMI on Difference in means
Difference in means
-18.50
-19.27
-20.04
-20.80
-21.57
-22.34
-23.11
-23.88
-24.64
-25.41
-26.18
24.18
24.44
24.71
24.97
25.24
25.50
25.76
26.03
26.29
26.56
26.82
B
Regression of HbA1c on Difference in means
Difference in means
-18.50
-19.27
-20.04
-20.80
-21.57
-22.34
-23.11
-23.88
-24.64
-25.41
-26.18
6.86
7.03
7.20
7.36
7.53
7.70
7.87
8.04
8.20
8.37
8.54
C
Regression of A ge on Difference in means
Difference in means
-18.50
-19.27
-20.04
-20.80
-21.57
-22.34
-23.11
-23.88
-24.64
-25.41
-26.18
55.14
55.69
56.24
56.80
57.35
57.90
58.45
59.00
59.56
60.11
60.66
Age
